# A finite element model for biomechanical characterization of ex vivo peripheral nerve dysfunction during stretch

**DOI:** 10.14814/phy2.70125

**Published:** 2024-11-13

**Authors:** Nicholas C. Vasas, Adam M. Forrest, Nathaniel A. Meyers, Michael B. Christensen, Jenny L. Pierce, Sidney M. Kaufmann, Kimberly B. Lanaghen, Randal C. Paniello, Julie M. Barkmeier‐Kraemer, Jonathan P. Vande Geest

**Affiliations:** ^1^ Department of Bioengineering, Swanson School of Engineering University of Pittsburgh Pittsburgh Pennsylvania USA; ^2^ Department of Otolaryngology – Head & Neck Surgery University of Utah School of Medicine Salt Lake City Utah USA; ^3^ Division of Urology, Department of Surgery University of Utah School of Medicine Salt Lake City Utah USA; ^4^ Department of Otolaryngology–Head and Neck Surgery Washington University School of Medicine St. Louis Missouri USA; ^5^ McGowan Institute for Regenerative Medicine University of Pittsburgh Pittsburgh Pennsylvania USA; ^6^ Vascular Medicine Institute University of Pittsburgh Pittsburgh Pennsylvania USA

**Keywords:** cauchy stress, compound action potential, finite element model, peripheral nerve damage, stretch

## Abstract

Peripheral nerve damage can cause debilitating symptoms ranging from numbness and pain to sensory loss and atrophy. To uncover the underlying mechanisms of peripheral nerve injury, our research aims to develop a relationship between biomechanical peripheral nerve damage and function through finite element modeling. A noncontact, ex vivo electrophysiology chamber, capable of axially stretching explanted nerves while recording electrical signals, was used to investigate peripheral nerve injury. Successive stretch trials were run on eight sciatic nerves (four females and four males) excised from Sprague–Dawley rats. Nerves were stretched until 50% compound action potential (CAP) amplitude reduction was obtained. A constitutive model developed by Raghavan and Vorp was suitable for rat sciatic nerves, with an average α and β of 0.183 MPa and 1.88 MPa, respectively. We then generated 95% confidence intervals for the stretch at which specific CAP amplitude reductions would occur, which compares well to previous studies. We also developed a finite element model that can predict stretch‐induced signaling deficits, applicable for complex nerve geometries and injuries. This relationship between nerve biomechanics and function can be expanded upon to create a clinical model for peripheral nerve dysfunction due to stretch.

## 1 INTRODUCTION

Early stages of peripheral nerve injury can cause sensory loss, numbness, pain, or burning sensations. Later stages of the disorder involve proximal numbness, distal weakening, or atrophy (Mayans & Mayans, 2015). To prevent adverse symptoms associated with peripheral nerve damage, it is pivotal to understand the mechanisms underlying peripheral nerve damage and dysfunction.

In characterizing peripheral nerve damage, Seddon initially described three categories, which were then expanded to six by Sunderland. The categories range from local demyelination to complete axonal lesion and connective tissue damage (Seddon, 1943; Sunderland, 1951). From these definitions of peripheral nerve damage and the experiments of Hodgkins and Huxley, who pioneered research on action potentials in a squid's giant axon, a path was paved to understand the interaction of biomechanical and electrophysiological properties in injured nerves (Hodgkin & Huxley, 1939).

After Huxley and Hodgkins evoked the first action potential using glass electrodes, more complex stimulation and recording techniques were invented (Hodgkin & Huxley, 1939). These techniques include a variety of noncontact and contact, in vivo and ex vivo stimulation and recording paradigms with various interfaces (suction electrodes, skin surface electrodes, sucrose gap, and AgCl electrodes) (Fritz Buchthal, 1996; Julian et al., 1962; Raymond, 1979; Stampfli, 1954; Stys et al., 1991). In these experiments, researchers either measured electrical activity from an individual neuronal fiber (action potential) or the summed electrical activity of many neurons (compound action potential). Compound action potentials (CAP)—defined by Stegeman as the summed electrical activity stemming from individual neuronal fibers, generated through stimulation of an entire nerve—could be readily obtained despite differences in recording modalities (Stegeman, 1981). Expanding upon these established recording and stimulating modalities, various experiments were conducted on the biomechanics and electrophysiology of peripheral nerves (Driscoll et al., 2002; Kwan et al., 1992; Li & Shi, 2007; Pelot et al., 2019; Rickett et al., 2011; Shi et al., 2000; Shi & Pryor, 2002; Xu et al., 2022).

During biomechanical nerve compression and stretch, studies have analyzed nerve morphology, including collagen fiber arrangement, demyelination, and axon integrity. Typically, greater demyelination, lower axon integrity, and collagen fiber straightening is seen under compression and stretch (Bianchi et al., 2018; Giannessi et al., 2020; Hewson et al., 2018; Kollech et al., 2022; Mahan et al., 2019; Smith et al., 1999; Sunderland & Bradley, 1961). Moreover, differences in local nerve rigidity have been seen across cranial and peripheral nerves alike (Phillips et al., 2004; Williams et al., 2015). Biological mechanisms, such as aortic arch compliance that may damage the left recurrent laryngeal nerve in idiopathic unilateral vocal fold paralysis, have been indicated to co‐occur with nerve dysfunction (Reza Behkam et al., 2017). Additionally, Shi and Pryor in their acute spinal nerve compression study have investigated nerve signaling in response to acute nerve injuries. These experiments found decreased CAP amplitude with greater nerve injury, focusing on finding the minimum injury where complete CAP amplitude recovery is possible (Kwan et al., 1992; Li & Shi, 2007; Rickett et al., 2011; Shi & Pryor, 2002; Xu et al., 2022). Emphasis has also been placed on decreased conduction velocity, perineurium and endoneurium resistivity, and altered Ca+ signaling after nerve damage (Driscoll et al., 2002; Pelot et al., 2019; Shi et al., 2000; Xu et al., 2022). An important component that was not a focus of these studies, however, is the development of a finite element model for stretch‐induced peripheral nerve injuries. Therefore, our analysis is specifically relevant for stretch‐induced injuries, such as traumatic injuries during collisions, but not for compression‐based injuries, such as those seen in diseases like carpal tunnel (Dubuisson & Kline, 2002; Han et al., 2011).

First, it is necessary to establish a relationship between peripheral nerve biomechanics and dysfunction. The rat sciatic nerve serves as a robust model that is commonly used for electrophysiology. Since it is a mixed nerve and the largest nerve in the rat, the sciatic nerve can be easily manipulated for electrophysiological experimentation (Giuffre et al., 2024). A threshold of 50% reduction in CAP amplitude, which was seen during greatest femoral lengthening in a study by Jou et al., can be set as a functional limit during biomechanical testing of the sciatic nerve (Jou et al., 2000). This is further supported by Kuntzer et al., who found that fewer than half of patients with axonal femoral neuropathy recovered after 1 year if compound motor action potential (CMAP) recordings experienced a 50% reduction (Kuntzer et al., 1997). Under these circumstances, implementing a noncontact electrophysiology recording chamber that is also capable of nerve stretching is prudent. This specific chamber design will allow the nerve to be stretched without incurring pathological damage due to friction between the nerve and recording interface.

Previously, Li and Shi used guinea pigs to develop an ex vivo noncontact peripheral nerve stimulation chamber that could record CAPs with stretch (Li & Shi, 2007). Rickett et al. expanded on this research to show the stress‐stretch and CAP amplitude recovery of peripheral nerves under specific loading in these animals (Rickett et al., 2011). This prior research was the first to utilize an ex‐vivo setup to investigate the relationship between peripheral nerve biomechanics and CAP amplitude. While the Rickett et al. manuscript reported stress‐stretch responses and alterations in CAP, these results were reported for guinea pigs on an averaged (across sample) basis and did not include a direct mapping between constitutive model parameters and CAP response.

Therefore, the purpose of this manuscript was to simultaneously collect electrophysiology and load from rat sciatic nerves exposed to increasing levels of mechanical stretch. These results were then used to develop specimen‐specific mechanical constitutive models that corresponded to known reductions in CAP. A finite element model of stretch‐induced peripheral nerve dysfunction, applicable to complex nerve geometries and injuries, was then established. Such information will be critical for the development of future constitutive modeling efforts that directly incorporate nerve dysfunction, thus opening new computational approaches to simulate both nerve dysfunction and in vivo mechanical deformation simultaneously.

## 2 MATERIALS AND METHODS

**FIGURE 1:**
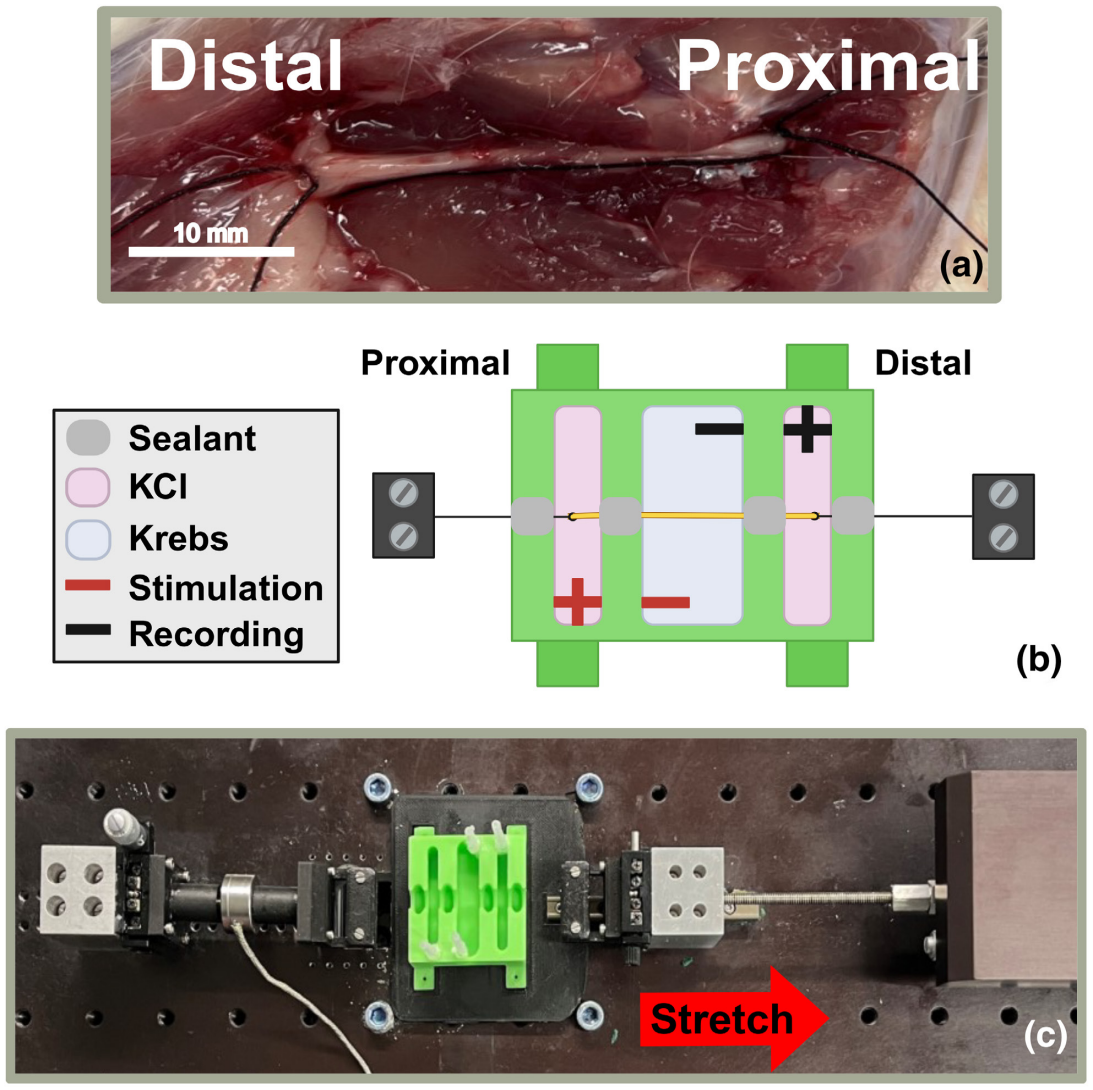
Sciatic nerve excision with electrophysiology chamber and uniaxial stretching device used for testing. (a) In situ sciatic nerve with sutures marking the bounds of the testing region. A long suture tie was used to indicate the distal end of the nerve, and a short suture tie for the proximal end. (b) Diagram of electrophysiology chamber used for stimulation and recording of the sciatic nerve. The proximal end of the nerve was stimulated, and CAPs were recorded from the distal end. The positive terminals were in wells containing 120 mM KCl solution, whereas the negative terminals were in a well containing Krebs‐ringer solution. Sealant was made of Vaseline, paraffin wax, and lanolin oil in a 3:1:1 ratio. Sealant was applied between solution wells and at the edges of the device to prevent solution mixing and leakage, respectively. (c) Uniaxial testing device with a 1 kg load cell attached for force recordings. Nerve was pulled to the right by a microslide stepper motor.

The Institutional Animal Care and Use Committee of the University of Pittsburgh approved all animal procedures. Four‐ to seven‐month‐old male and female Sprague–Dawley rats (Envigo) weighing 359 g ± 75 g were euthanized with carbon dioxide asphyxiation and cervical dislocation. Immediately following, the sciatic nerve was excised after tying one 0.3 mm silk suture (Fisher Scientific, 10000658) proximal to the peroneal and tibial branches, and another suture distal to the spinal cord nerve root (Figure 1a). To ensure correct stimulation orientation, a longer suture was tied to the distal end of the nerve and a shorter suture to the proximal end. Nerve excision took less than 25 min to complete. The nerve was transferred to an hour‐long incubation in bicarbonate buffered Krebs‐ringer solution (ThermoFisher Scientific, J67591‐K2), oxygenated to maintain a pH between 7.2 and 7.4 as measured by a pH probe (Fisher Scientific, 01912346). After incubation, the length between suture ties was measured for baseline length. An average nerve length of 28.8 mm, with a range of 24.7 mm–35.2 mm, was obtained. Four right nerves (two male and two female) and four left nerves (two male and two female) were stimulated, with the contralateral nerve of each animal excised to serve as a non‐stretched histological control.

### 2.1 CAP recording

The methods for CAP recording were adapted from the noncontact, ex vivo electrophysiology chamber designed by Li and Shi (2007). After oxygenation, nerves were placed in a 3D printed stimulation chamber outlined in Figure 1b. The nerve was placed in the stimulation chamber with sealant surrounding the nerve between the different sections of the solution wells. Sealant was made by combining Vaseline, paraffin wax, and lanolin oil in a 3:1:1 ratio at 200°C and then letting it cool to room temperature. Careful consideration was made to ensure a portion of the nerve between the sutures was in all the solution wells. The inner solution well was filled with Krebs‐ringer solution to provide the necessary external environment for compound action potential production (Thermo Scientific, J67591.K2). By percentage weight, Krebs‐ringer solution contains 98.92% water, 0.701% sodium chloride, 0.21% sodium bicarbonate, 0.099% glucose, 0.037% potassium chloride, 0.022% calcium chloride, and 0.01% magnesium chloride. The outer wells were filled with 120 mM KCl to prevent action potentials and serve as a reference recording. KCl was dyed green with food coloring in preliminary experiments to ensure no leakage between the outer and inner solution wells.

**FIGURE 2:**
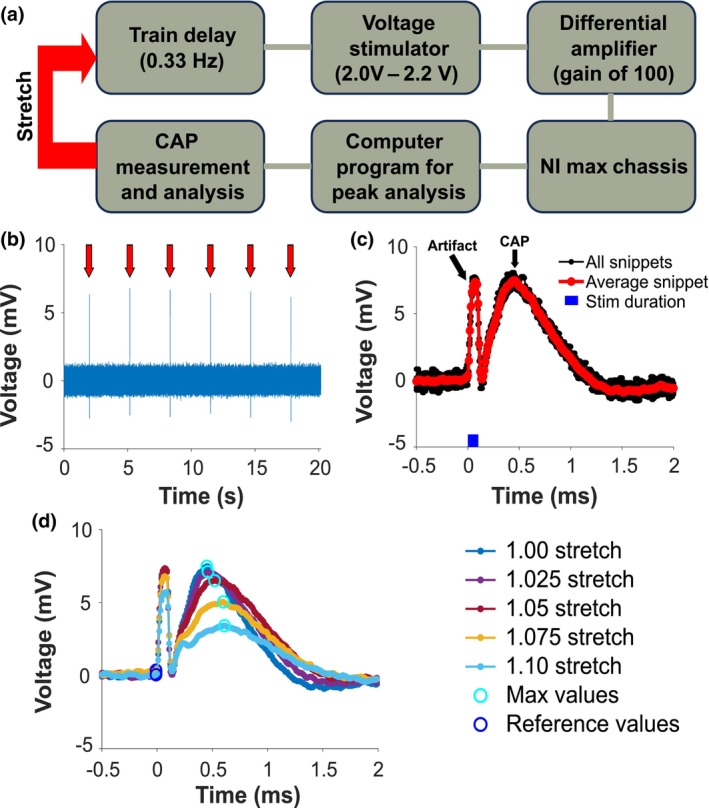
Increased stretch of rat sciatic nerves reduces CAP amplitude. (a) Stimulation and recording paradigm for each nerve trial. (b) Identifying CAP peaks over the 20 s of recording after each stretch test. Individual CAP peaks are highlighted with red arrows. (c) Snippets of raw CAPs (black) versus average CAP (red) across one stretch test. Stimulation duration of 100 μs is identified in blue, while black arrows identify CAP and artifact peaks. (d) Decrease in average CAP amplitude with increasing stretch. Peak CAP and baseline voltage values were manually approximated using getpts to calculate a percent reduction in CAP amplitude.

Ag/AgCl 0.5 mm electrodes (World Precision Instruments, EP05) were vertically placed in Krebs‐ringer and KCl wells through nonconducting plastic guides (the exact position can be seen in Figure 1c). Before stimulation, the sutures were tied to each grip of the uniaxial stretching device and pulled until the sample was taught. The uniaxial stretching device (Figure 1c) consists of a microslide stepper motor (Newmark Systems, MS‐2‐24), motion controller (Newmark Systems, NSC‐1S), and a 1‐kg load cell (Omega, LCFL‐1KG). Stimulation was provided at the proximal end of the nerve, utilizing a train delay (Digitimer, DG2A) and voltage stimulator (Digitimer, DS2A). The nerves were stimulated at 0.33 Hz for about 20 s, with stimulus amplitude ranging from 2.0 V to 2.2 V and duration between 40 μs and 100 μs. Stimulation amplitude was altered to create maximum CAP amplitudes, while duration was changed to prevent temporal overlap of the recorded CAP and stimulus artifact. Recording terminals were grounded in Krebs‐ringer solution and sent to a differential amplifier (Warner Instruments, DP‐304A), amplified with a gain of 100. The signal was preprocessed with an analog band‐pass filter between 10 Hz and 5000 Hz. At a sampling rate of 100,000 Hz, the signal was transmitted to an NI max DAQ chassis (National Instruments, cDAQ‐9188) through a voltage input module (National Instruments 193299G‐01L). The signal was then digitally interpreted in MATLAB. No evident CAP was recorded from the third male tested, so it was excluded from analysis. The stimulation and recording flow diagram is outlined in Figure 2a.

### 2.2 CAP under stretch

After obtaining a CAP by adjusting stimulation amplitude and duration, the nerve was stretched using the uniaxial device. Nerves were stretched at a speed of 0.1 mm/s with percentage stretches of 1.000, 1.025, 1.050, 1.075, 1.100, 1.125, 1.150, 1.175, and 1.200. CAP recordings started immediately after each stretch level was obtained. Nerves were stretched until 1.20 stretch or 50% reduction in CAP amplitude, whichever occurred first. The individual stretch and stimulation tests took on average 5 min to complete, with the total time of stimulation after oxygenation not exceeding 2 h. A sample of raw CAP recordings are highlighted with red arrows in Figure 2b. A digital band‐pass filter of 100 Hz–5000 Hz was applied to CAP recordings to remove line noise. The CAPs were additionally baseline subtracted by averaging datapoints 5 ms before stimulation and median filtered using a 100 μs sliding window. A plot of the individual CAPs (black) and average CAP (red) for a stimulation sequence in the second male tested at 1.00 stretch can be seen in Figure 2c.

Following 1.00 stretch (unstretched) CAP recordings, stretch tests commenced. For each stretch test, force recordings required to reach each stretch were received by the load cell and transferred to a terminal block (National Instruments, 150888A‐01L) in the DAQ chassis for post hoc interpretation. After a stretch test was completed, stimulation and CAP recording commenced. Average CAP amplitude and baseline voltage values were selected using the manual getpts function in MATLAB. CAP amplitude percent reduction, R_n_, was calculated using Equation 1, where n is the stretch increment, C_1.00_ represents CAP amplitude at 1.00 stretch, and C_n_ is CAP amplitude with stretch n.

(1) $$R_{n}=\frac{C_{1.00}-C_{n}\;}{C_{1.00}}*100\%$$

For every stretch trial, a percent reduction in CAP amplitude was recorded until at least a 50% reduction in CAP amplitude was observed or 1.20 stretch was reached. A sample of successive stretch trials on the second male's nerve can be seen in Figure 2d. Nerves were prepared for collagen angle analysis following stretch trials.

To prepare for nerve fixation, Krebs‐ringer and KCl solutions were removed from their respective wells, while sealant was removed from the inner sections separating Krebs‐ringer and KCl. Krebs‐ringer and KCl were replaced with 4% paraformaldehyde (PFA) to fix the nerve at the stretch level producing 50% CAP amplitude reduction. After at least 10 min soaking in 4% PFA, the nerve was removed from the uniaxial device and stimulation chamber. The nerve was fixed overnight in 4% PFA and stored in a 1% phosphate‐buffered saline (PBS) solution with 0.1% sodium azide. Furthermore, the contralateral nerve was fixed at its post‐incubation length, utilizing the same procedure as the stretched nerve.

### 2.3 Second harmonic generation imaging

Collagen angle imaging from stretched and non‐stretched nerves were conducted as previously described (Kollech et al., 2022). Briefly, all nerves were equilibrated in a 30% sucrose solution before sectioning on a Leica CM1950 cryostat (Leica, Bannockburn, IL) at 100 μm thickness. Three sections per nerve were used for quantification, these being located approximately ¼, ½, and ¾ along the depth of the nerve. Type I collagen fibers were visualized via second harmonic generation (SHG) imaging using a Bruker Ultima multi‐photon confocal microscope with a Nikon LWD 16× water immersion objective (numerical aperture of 0.8) and Prairie View Software. A Coherent Chameleon Vision IR laser was tuned to a wavelength of 800 nm, and detection was performed using non‐descanned PMT detectors. SHG signals were discriminated from background autofluorescence using 460LP dichroic and 377/50 nm band‐pass filters. XY pixel size was 0.81 × 0.81 μm, with a 5 μm z‐step size.

### 2.4 Data analysis

Data analysis was performed to investigate the material properties (stress‐stretch) and function (CAP) of rat sciatic nerves under stretch. To develop a relationship between mechanical response and percent reduction in CAP amplitude, a spline was fit to each trial using the csaps function in MATLAB. Interpolating from the spline, stretch values for every 2.5% CAP amplitude reduction were obtained. These intervals were utilized to develop 95% confidence intervals for the stretch needed to produce a specific CAP amplitude reduction.

To develop a stress‐stretch relationship, precise diameter measurements and force readings were obtained. Force data was acquired by taking the maximum force from each stretch increment. Diameter measurements were obtained from post‐oxygenated nerve images by averaging five diameter measurements across the length of the nerve. Cauchy stress, T_11_, was calculated at each stretch according to Equation 2.

(2) $$T_{11}=\left(\frac{f_{1}}{A_{o}}\right)\lambda_{1}$$

Here, f_1_ is the force to produce a stretch, A_o_ is the initial cross‐sectional area (assumed to be circular), and λ_1_ is a stretch increment. Following the work of Raghavan and Vorp, as well as the interpretation of Williams et al., the sciatic nerve material properties α and β (units of MPa) were fit to the constitutive model represented by Equation 3 (Raghavan & Vorp, 2000; Williams et al., 2015).

(3) $$T_{11}=\left[2\alpha +4\beta \left(\lambda_{1}^{2}+2\lambda_{1}^{-1}-3\right)\right]\left[\lambda_{1}^{2}-\lambda_{1}^{-1}\right]$$

This analysis allowed for collection of Cauchy stress versus stretch and CAP alterations in all rat trials.

Latency, another indicator of nerve function, is the delay in CAP peak response relative to stimulation. Utilizing the time difference between onset of stimulation and average CAP peak, latency was calculated for all stretch increments across all trials. The manual getpts function in MATLAB was used to obtain latency values from CAP peaks. Latency values were compared for unstretched and stretched trials when a 50% CAP amplitude reduction occurred.

Collagen fiber angles were quantified using a custom MATLAB code (Ram et al., 2018; Sander & Barocas, 2009). Fiber angles were normalized so that the mean fiber angle for each section was equal to zero (the assumption being that the mean direction along the length of the nerve would be equal to zero). Fiber angles for each nerve were binned into 1° increment histograms.

Finite element modeling allows the future utility of the developed mapping relationships (stress‐stretch‐CAP reduction) for more complex (e.g., patient specific) anatomical geometries across any number of different clinical applications. To demonstrate the utility of mapping constitutive (stress‐stretch) relationships, a simple finite element model of one experiment was constructed and run using the open‐source software FEBio. The isotropic hyperelastic model described above was implemented in FEBio and a mapping was generated in Matlab (version R2023b) where local stretch values were linearly interpolated to generate CAP reduction values. Sample specific geometries and axial stretches (for a representative sample) were imposed on this model as a demonstrative application.

### 2.5 Statistical analysis

In MATLAB, a two‐way ANOVA was run to compare sex (male versus female) and side (left versus right) for stretch at 50% reduction in CAP amplitude. A two‐sample student's *t*‐test was also run to test if there was a significant difference between latency for unstretched tests and tests when a 50% CAP amplitude reduction occurred. Interpolating from a spline for each nerve, 95% confidence intervals were calculated for stretch levels from 0% reduction to 50% reduction in intervals of 2.5% reduction. Additionally, r‐squared and coefficient analyses were collected from Cauchy stress‐stretch data. To test for differences in sidedness and sex in constitutive modeling, a two‐way ANOVA was run for alpha and beta individually. This analysis serves to confirm a link between the biomechanical properties and functional output of the sciatic nerve.

Fiber angle analysis was completed for each nerve by identifying the histogram bin with the highest percentage of fibers. Full width at half maximum (FWHM) of the fiber angle distribution was defined as the width of the distribution when it crosses half the maximum percentage of fibers. FWHM values were used for statistical analysis of fiber angle distributions. One of the stretched nerve samples was excluded from this analysis, as it was identified as an outlier based on interquartile testing (outliers were less than Q1‐1.5*IQR or greater than Q3 + 1.5*IQR). An F‐test was used to determine if the variances in the stretched and non‐stretched groups were similar, and a student's *t*‐test was used to compare FWHM values between groups. A *p* value <0.05 was considered significant.

## 3 RESULTS

**FIGURE 3:**
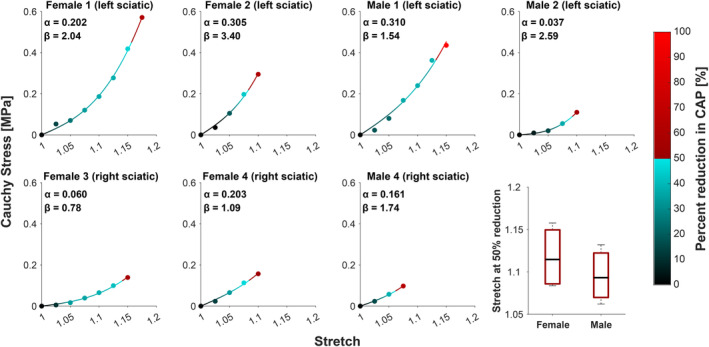
Cauchy stress‐stretch plots showing the stretch corresponding to at least 50% CAP amplitude reduction in each nerve. The color gradient represents CAP amplitude reduction, where 50% reduction or greater is highlighted in red. The minimum stretch at 50% CAP amplitude reduction across all trials is 1.075, with a maximum of 1.175. Differences between male and female stretch at 50% reduction (approximated from fitting a spline) were not significant (two‐way ANOVA, *p* = 0.41).

The third male nerve was excluded from analysis due to likely surgical damage, resulting in recordings with unidentifiable CAP. The rest of the sciatic nerves produced evident CAPs with stimulation. User error in CAP amplitude acquisition was estimated to be 1.04% based on two additional trials of the manual getpts function. Results of a two‐way ANOVA indicated no significant difference between stretch at 50% CAP amplitude reduction and sex (*p* = 0.41), side (*p* = 0.44), or sex and side interaction (*p* = 0.54). For all seven nerves, stress‐stretch curves were approximated following the work of Raghavan and Vorp (Equation 3) (Raghavan & Vorp, 2000). The color gradient values reflected percent reduction in CAP amplitude fit to a spline (Figure 3). Across the seven nerves tested, the average measured stretch to achieve at least a 50% reduction in CAP amplitude was 1.11 with a range of 1.075–1.15. The average percent reduction where stretch tests were concluded was 63.91%, with the first male seeing 100% reduction as the CAP completely disappeared. The percent reduction values across all trials are presented in Table A1.

**FIGURE 4:**
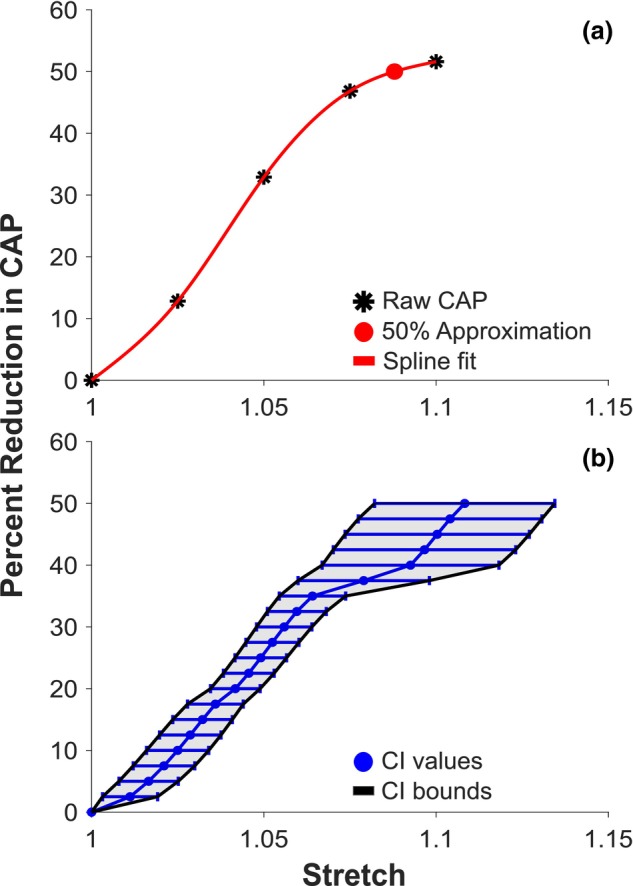
The 95% confidence intervals used to develop a relationship between CAP amplitude reduction and stretch. (a) Sample from the fourth female. A spline (red) was used to approximate raw CAP amplitude reduction (black asterisks). Denoted with a red marker is the interpolated stretch value where 50% CAP amplitude reduction occurs. (b) The 95% confidence intervals at specified CAP amplitude reductions for all seven trials (blue). The shaded area (light gray) represents the interval between the lower and upper bounds (black) of all 95% confidence intervals.

Representative raw data, spline estimation, and interpolated point of 50% reduction in CAP amplitude from the fourth female stretch trials can be seen in Figure 4a. The 95% confidence interval for stretch with CAP amplitude reduction was calculated at 2.5% segments between 0% and 50% CAP amplitude reduction (Figure 4b). The lower and upper bounds of the 95% confidence interval increased with higher percent reductions with a CI for stretch at 50% CAP amplitude reduction as 1.108 ± 0.026. The specific 95% confidence intervals for each percent reduction in CAP amplitude can be found in Table A2. When running two additional trials with the manual getpts software to obtain latency values for the first male, a user error of 2.00% was obtained. A two‐sample student's *t*‐test was run and found no significant difference between latency during trials where the nerve was unstretched and trials where testing concluded (*p* = 0.39).

**FIGURE 5:**
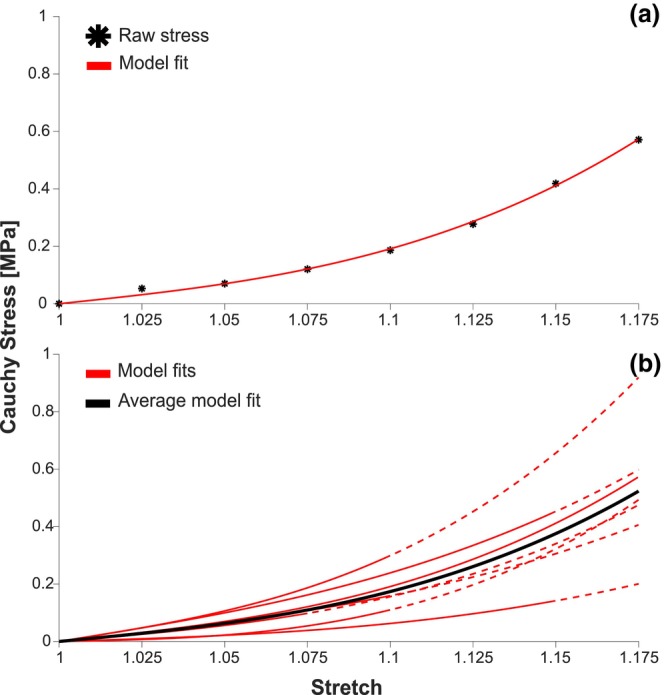
Constitutive modeling for all nerves. (a) Raw Cauchy stress for each stretch (black asterisks), plotted with a constitutive model approximation using Equation 3 (red) for the first female. (b) Constitutive model shown for each nerve (red), as well as the average across all trials (black, average α = 0.183 MPa, average β = 1.88 MPa). The dashed line represents periods where no stretch was induced and only extrapolated from the model.

An average nerve diameter of 1.42 mm and a range of 1.30 mm–1.53 mm were measured. Biomechanical modeling of the nerves, following the work of Raghavan and Vorp (Equation 3), produced a range of *r*‐squared values between 0.99 and 1.00, with an average of 1.00 (Raghavan & Vorp, 2000). A sample fit from the first female can be seen in Figure 5a. A range of α values from 0.037 to 0.31, and β values from 0.78 to 3.40 were obtained, with averages of 0.183 and 1.88, respectively (Table 1). A maximum raw Cauchy stress of 0.57 MPa was seen across all nerves. An overlay of individual trial estimations and average α and β fit can be seen in Figure 5b. Cauchy stress modeling was extrapolated for stretch values beyond where testing concluded until a stretch of 1.175. A two‐way ANOVA for α showed no significant differences in sex (*p* = 0.82), side (*p* = 0.57), and sex and side interactions (*p* = 0.64); the two‐way ANOVA for β also showed no significant difference in sex (*p* = 0.90), side (*p* = 0.16), and sex and side interactions (*p* = 0.29). Cauchy stress values, used for constitutive modeling across all nerves, can be seen in Table A3.

**TABLE 1:** Approximated α and β values for stress‐stretch constitutive modeling, calculated from Equation 3.

Trial	Alpha [MPa]	Beta [MPa]
Female 1	0.202	2.04
Female 2	0.305	3.40
Female 3	0.060	0.78
Female 4	0.203	1.09
Male 1	0.310	1.54
Male 2	0.037	2.59
Male 4	0.161	1.74
Average	0.183	1.88

**FIGURE 6:**
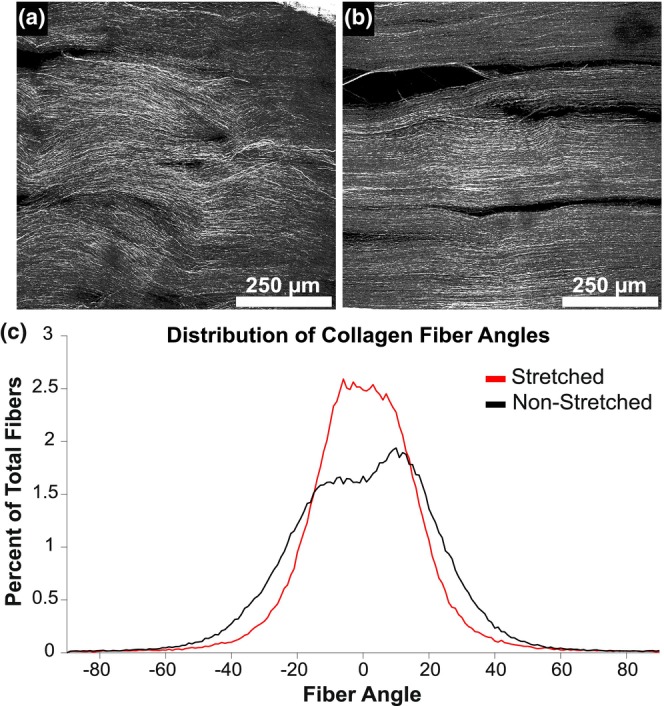
SHG imaging and analysis of non‐stretched and stretched nerves. (a) Representative SHG image of non‐stretched nerves. (b) Representative SHG image of stretched nerves. (c) Distribution of average collagen fiber angles for both groups. Analysis of FWHM data revealed significant differences between groups (*p* = 0.005).

Representative images of non‐stretched and stretched nerves are shown in Figure 6a,b, respectively. Analysis of FWHM data revealed significant differences between groups (*p* = 0.005), with fibers from the stretched group having a tighter distribution (Figure 6c).

**FIGURE 7:**
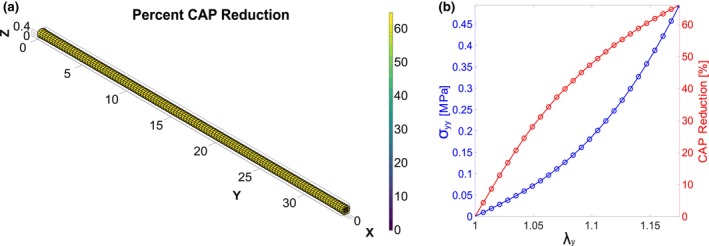
Demonstrative simulation of CAP reduction using finite element modeling. (a) Final deformed geometry of finite element model for a representative experimental sample (legend is CAP reduction). (b) Axial stress and CAP reduction from the finite element model utilizing the sample from panel a.

Using a representative sample from rat sciatic stretch trials, we depicted the final, deformed nerve geometry as a function of CAP amplitude reduction through finite element modeling (Figure 7a). We then developed a relationship between axial stress, stretch, and CAP reduction from our sample‐specific finite element model (Figure 7b).

## 4 DISCUSSION

From this work, we were able to develop a relationship between CAP and a mechanical constitutive model for the rat sciatic nerve. Despite previous reports demonstrating sided differences in myelin thickness in the rat sciatic nerve, we found no significant difference in constitutive modeling parameters or average stretch at 50% CAP amplitude reduction between sex, side, or sex and side interactions (*p* > 0.05) (Christensen & Tresco, 2015). Additionally, no significant difference in latency was found between unstretched tests and final stretch tests. The 95% confidence intervals produced a relationship between CAP amplitude reduction and stretch. A 95% confidence interval of 1.108 ± 0.026 stretch was observed at a 50% CAP amplitude reduction. Furthermore, constitutive modeling of Cauchy stress versus stretch across all seven stretch trials produced average α and β values for the constitutive model of 0.183 MPa and 1.88 MPa, respectively. Our constitutive model produced a maximum raw Cauchy stress of 0.57 MPa for 1.175 stretch. Analysis of collagen fiber angles showed a significant alignment of fibers along the length of the nerves in the stretched group. Implementation of a finite element model for a representative rat sciatic nerve showed changes in CAP reduction and axial stress with stretch, which can be applied to complex nerve geometries.

While investigating CAP amplitude recovery with stretch in peripheral nerves, Li and Shi found that a 50% CAP amplitude reduction in guinea pig nerves occurs at a stretch level of 1.10 (Li & Shi, 2007). This stretch value falls within the 95% confidence interval we calculated (1.108 ± 0.026). This is significant because it supports the consistency in the device's ability to cause similar functional deficits with stretch across species. Moreover, an in vitro study of rabbit sciatic nerves by Kwan et al. showed that 12% strain is sufficient to reduce CAP amplitude by 50%. Again, this 12% strain falls within our calculated confidence interval for 50% CAP amplitude reduction (Kwan et al., 1992). Rickett et al. found that CAP amplitude recovered to 58 ± 6% of baseline recordings at 15% strain in guinea pigs (Rickett et al., 2011). Although it would seem their results are inconsistent with the CAP‐stretch confidence interval outlined in this paper, they recorded CAP recovery and not initial reductions. Their recordings show that CAP amplitude initially plunges below 50% of baseline at 15% strain. Therefore, a strain around 15% may have initially produced a 50% reduction in CAP amplitude, but the precise stretch value was not reported. Additional studies have confirmed the correlation between greater stretch or compression and increased CAP amplitude reduction (Shi & Pryor, 2002; Xu et al., 2022).

Previous experiments measuring the biomechanical properties of peripheral nerves have found Cauchy stress for nerves on the order of MPa. Estimated maximum Cauchy stress values of 5 MPa for 20% strain, 9 MPa for 32% strain, and 12 MPa for 40% strain were observed across in vitro and ex vivo studies alike (Kwan et al., 1992; Li & Shi, 2007; Rydevik et al., 1990). These values are slightly higher than what was observed in our study (0.57 MPa at 1.175 stretch), which could be attributed to the fact that they tested higher stretch levels. It has been clearly indicated in tensile loading experiments that high stretch levels increase axonal permeability, decrease lamellae distance in myelin, pucker perineurium, instigate perineurium damage, straighten nerve fibers, and induce axonal thinning (Bianchi et al., 2018; Denny‐Brown & Doherty, 1945; Driscoll et al., 2002; Haftek, 1970; Hoen & Brackett, 1956; Smith et al., 1999). All these factors, or one of these factors individually, could be responsible for the decrease in CAP amplitude observed in our experiments. While other studies demonstrated blood vessel compression and decreased blood flow in response to tensile loading, it is not relevant in our study because all CAP measurements were taken ex vivo (Denny‐Brown & Doherty, 1945; Driscoll et al., 2002; Haftek, 1970; Jou et al., 2000). This suggests that decreases in blood flow would likely further exacerbate the mechanically induced CAP reduction observed in our study.

Fixation and analysis of nerves at stretch levels that resulted in 50% CAP amplitude reduction demonstrated an alignment of fibers along the length of the nerve. This finding is in line with other studies that have investigated collagen fiber angles in response to nerve stretch (Bianchi et al., 2018; Kollech et al., 2022). Although it is unclear if collagen fiber alignment directly correlates with nerve function, alignment of these fibers would suggest a transfer of mechanical stress onto epineural collagen. An understanding of the degree to which the angle distribution of these fibers tightens before mechanical damage to underlying nerve fibers occurs may shed further light into the mechanisms of CAP disruption and warrants further investigation to determine the limit between passive and more permanent injury. In future studies, staining for axon biomarkers using Tuj1 or SCG10 would aid in understanding the extent that stress associated with collagenous straightening disrupted signaling capabilities of the nerve fiber.

An important limitation of our study was that stimulation and recording techniques were conducted indirectly and ex vivo. This means that the signal sent for stimulation of the nerve and received for CAP recordings had to be transmitted through solution. Transmission through solution likely increased variability of stimulation reaching the nerve and lowered signal‐to‐noise ratio during CAP recordings. Moreover, successive stretch trials being run on the same nerve could have compounded damage, resulting in a more abrupt decrease in CAP than what may be seen in stretch‐relaxation experiments. In this study, nerves were held at each stretch interval for 1 min on average. In future experiments, we can assess how prolonged time at stretch with successive stretches on the same nerve would impact signaling deficits and the viscoelastic and viscoplastic properties of the nerve. Additionally, the stretch rate of 0.1 mm/s could be varied to better understand how the rate of injury affects signaling deficits. Our experimental design also prevented within‐subject comparisons between left and right nerves, which would have provided more robust statistical comparisons. Further analysis of our data shows that the Cauchy stress required to reach 1.075 stretch for left and right nerves is 0.135 MPa ± 0.062 and 0.083 MPa ± 0.039, respectively. The larger average Cauchy stress required for left nerves to reach 1.075 stretch may be due to myelination differences in sidedness, although not significant here (Christensen & Tresco, 2015).

Even with these drawbacks, this study provides the next step towards more complex modeling of the relationship between peripheral nerve biomechanics and function. This relationship could aid current research focused on developing allografts, autografts, and stimulation techniques to alleviate peripheral nerve damage and associated pain (Clint Hansen et al., 2016; Gordon, 2016; Wangensteen & Kalliainen, 2010). Specifically, we showed a straightening of collagen fibers with increased stretch, which could serve as a conduit for topological designs to mimic the natural biomechanical microenvironment, improving cell migration, rearrangement, and attachment. All these factors could accelerate axonal regeneration in stretched nerves. Furthermore, collagen straightening impacts extracellular mechanotransduction pathways, which dynamically maintain cell proliferation, differentiation, and maturation (Kong et al., 2022). Additionally, inside a silk conduit, silk nanofiber fillers with hierarchical anisotropic structures can be used to carry various cargo/signaling molecules (Lu et al., 2021). Imbedding a collagenous network in these silk nerve conduits would ensure both an adequate elastic modulus and improved signaling for better support in long‐term in vivo nerve repair (Kong et al., 2022). Understanding how stretch impacts both ECM components and CAP signaling at critical deficits (50% CAP reduction) would allow for complex and necessary in vivo mimicry for nerve conduit scaffolds.

In this work, we have quantified with high confidence a finite element model for the relationship between CAP amplitude reduction and biomechanical response of rat sciatic nerves observed ex vivo. This research serves as a foundation for future computational models simulating peripheral nerve function in response to specific in vivo deformations and stretch injuries, such as trauma, but not for compression injuries, like carpal tunnel. In future studies, we can then understand local nerve rigidity and consider the complexity of surrounding morphology in finite element modeling.

## AUTHOR CONTRIBUTIONS

N.C.V., A.M.F., N.A.M., B.M.C., R.C.P., J.M.B.‐K., and J.P.V.G. conceived and designed research; N.C.V., S.M.K., J.L.P., and M.B.C. performed experiments; N.C.V., A.M.F., K.B.L., M.B.C., and J.P.V.G. analyzed data; N.C.V., A.M.F., M.B.C., and J.P.V.G. interpreted results of experiments; N.C.V., A.M.F., M.B.C., and J.P.V.G. prepared figures; N.C.V. drafted manuscript; N.C.V., A.M.F., N.A.M., K.B.L., S.M.K., J.L.P., M.B.C., R.C.P., J.M.B.‐K., and J.P.V.G. edited and revised manuscript; N.C.V., A.M.F., N.A.M., K.B.L., S.M.K., J.L.P., M.B.C., R.C.P., J.M.B.‐K., and J.P.V.G. approved final version of manuscript.

## FUNDING INFORMATION

Funding for this research was provided by the National Institute on Deafness and Other Communication Disorders (Grant 5‐R01‐DC‐011311 to J. M. Barkmeier‐Kraemer and J. P. Vande Geest). Funding was also provided by the Swanson School of Engineering and the Office of the Provost at the University of Pittsburgh through the summer undergraduate research internship (SURI) program.

## CONFLICT OF INTEREST STATEMENT

No conflicts of interest, financial or otherwise, are declared by the authors.

## ETHICS STATEMENT

All animal studies at Pitt were conducted in accordance with the Public Health Service Policy on Humane Care and Use of Laboratory Animals.

## Data Availability

The data that support the findings of this study are available from the corresponding author upon reasonable request.

## Appendix

**TABLE A1:** Percent reduction in CAP amplitude (Equation 1) for the seven rat sciatic trials. A positive reduction represents a decrease in CAP amplitude, while a negative reduction shows an increase in CAP amplitude. If no evident CAP was present after a stretch trial, CAP amplitude reduction was set to 100%. Trials were run until 50% reduction in CAP or 1.20 stretch was obtained for a given stretch level.

Stretch	Female 1 (left sciatic) CAP reduction [%]	Female 2 (left sciatic) CAP reduction [%]	Female 3 (right sciatic) CAP reduction [%]	Female 4 (right sciatic) CAP reduction [%]	Male 1 (left sciatic) CAP reduction [%]	Male 2 (left sciatic) CAP reduction [%]	Male 4 (right sciatic) CAP reduction [%]
1.000	0.00	0.00	0.00	0.00	0.00	0.00	0.00
1.025	14.55	−1.23	10.46	12.82	17.37	5.07	15.07
1.050	24.71	18.21	33.32	32.90	21.16	14.79	37.63
1.075	33.39	43.32	34.87	46.83	36.38	36.59	63.61
1.100	36.50	59.69	37.50	51.60	37.63	54.43	–
1.125	37.58	–	40.79	–	38.35	–	–
1.150	45.65	–	56.43	–	100	–	–
1.175	61.59	–	–	–	–	–	–

**TABLE A2:** The 95% confidence intervals for stretch given a CAP amplitude reduction. Interpolation from a fit spline allowed the calculation of 95% confidence intervals for stretch given a CAP amplitude reduction. The 95% confidence intervals for stretch were calculated at 2.5% CAP reduction segments. Data are represented as mean ± variation.

Percent CAP reduction	Stretch confidence interval
0.0	1.000 ± 0.000
2.5	1.011 ± 0.008
5.0	1.017 ± 0.009
7.5	1.021 ± 0.009
10.0	1.025 ± 0.009
12.5	1.029 ± 0.009
15.0	1.032 ± 0.009
17.5	1.036 ± 0.008
20.0	1.042 ± 0.007
22.5	1.046 ± 0.007
25.0	1.049 ± 0.008
27.5	1.053 ± 0.008
30.0	1.056 ± 0.008
32.5	1.060 ± 0.009
35.0	1.064 ± 0.010
37.5	1.079 ± 0.019
40.0	1.093 ± 0.026
42.5	1.097 ± 0.027
45.0	1.100 ± 0.027
47.5	1.104 ± 0.027
50.0	1.108 ± 0.026

**TABLE A3:** Cauchy stress for rat sciatic stretch trials. Data represents Cauchy stress [MPa] for each stretch interval. Cauchy stress was calculated according to Equation 2.

Stretch	Female 1 (left sciatic) stress [MPa]	Female 2 (left sciatic) stress [MPa]	Female 3 (right sciatic) stress [MPa]	Female 4 (right sciatic) stress [MPa]	Male 1 (left sciatic) stress [MPa]	Male 2 (left sciatic) stress [MPa]	Male 4 (right sciatic) stress [MPa]
1.000	0.000	0.000	0.000	0.000	0.000	0.000	0.000
1.025	0.053	0.036	0.005	0.024	0.023	0.009	0.023
1.050	0.070	0.105	0.017	0.066	0.080	0.020	0.058
1.075	0.120	0.197	0.039	0.113	0.168	0.055	0.098
1.100	0.187	0.295	0.065	0.157	0.240	0.110	–
1.125	0.278	–	0.100	–	0.362	–	–
1.150	0.419	–	0.139	–	0.436	–	–
1.175	0.571	–	–	–	–	–	–
